# Hospitalized acute exacerbation in chronic obstructive pulmonary disease – impact on long-term renal outcomes

**DOI:** 10.1186/s12931-023-02635-8

**Published:** 2024-01-18

**Authors:** Wang Chun Kwok, Terence C. C. Tam, James C. M. Ho, David C. L. Lam, Mary S. M. Ip, Desmond Y. H. Yap

**Affiliations:** 1 Division of Respiratory Medicine, The University of Hong Kong, Hong Kong SAR, People’s Republic of China; 2 Division of Nephrology, Department of Medicine, Queen Mary Hospital, the University of Hong Kong, Hong Kong Special Administrative Region, Hong Kong SAR, People’s Republic of China

**Keywords:** Acute exacerbation, Acute kidney injury, Chronic Obstructive Pulmonary Disease, Chronic Kidney Disease

## Abstract

**Introduction:**

Acute exacerbation of chronic obstructive pulmonary disease (AECOPD) is a common and preventable event in patients with chronic obstructive pulmonary disease (COPD). Data regarding the impact of AECOPD on short- and long-term renal outcomes are lacking.

**Methods:**

We included all COPD patients who were followed at Queen Mary Hospital (QMH) in year 2015 and reviewed their clinical/renal outcomes in subsequent five years. Relationships between AECOPD and adverse renal outcomes were evaluated.

**Results:**

371 COPD patients were included. 169 patients had hospitalized AECOPD in past one year (HAE group) while 202 patients did not (non-HAE group). 285 patients (76.8%) had renal progression/death and 102 (27.5%) patients developed acute kidney injury (AKI). HAE group showed a more rapid eGFR decline than non-HAE group (-4.64 mL/min/1.73m^2^/year vs. -2.40 mL/min/1.73m^2^/year, *p* = 0.025). HAE group had significantly higher risk for renal progression/death at 5 years [adjusted OR (aOR) 2.380 (95% CI = 1.144–4.954), *p* = 0.020]. The frequency of hospitalized AECOPD in past 3 years, any AECOPD in past 3 years, hospitalized AECOPD in past 3 years were also predictive of renal progression/death at 5 years [aOR were 1.176 (95% CI = 1.038– 1.331), 2.998 (95% CI = 1.438–6.250) and 2.887 (95% CI = 1.409–5.917) respectively; *p* = 0.011, 0.003 and 0.004]. HAE group also showed significantly higher risk of AKI [adjusted HR (aHR) 2.430; 95% CI = 1.306–4.519, *p* = 0.005].

**Conclusions:**

AECOPD, in particular HAE, was associated with increased risk of renal progression/death and AKI. Prevention of AECOPD, especially HAE, may potentially improve short- and long-term renal outcomes in COPD patients.

**Supplementary Information:**

The online version contains supplementary material available at 10.1186/s12931-023-02635-8.

## Summary at a glance

Acute exacerbation of chronic obstructive pulmonary disease (AECOPD), in particular hospitalized acute exacerbation (HAE), was associated with increased risk of renal progression/death and acute kidney injury. Prevention of AECOPD, especially HAE, may potentially improve short- and long-term renal outcomes in COPD patients.

**Supplementary Information:**

The online version contains supplementary material available at 10.1186/s12931-023-02635-8.

## Introduction

Acute exacerbation of chronic obstructive pulmonary disease (AECOPD) confers excess morbidity and mortality [1–3]. Chronic obstructive pulmonary disease (COPD) per se is associated with adverse clinical outcomes in other organ systems. The relationship between COPD and chronic renal impairment is intriguing [4]. Previous studies suggested that AECOPD patients had relatively high incidence and prevalence of acute kidney injury (AKI) [5–7]. COPD patients with AKI during acute exacerbations also had more severe COPD and higher healthcare utilization [7–10]. Apart from AKI, COPD patients showed high prevalence of CKD, up to 43% [11]. The presence of concurrent COPD also increases the risk of mortality amongst advanced CKD patients, and yet COPD per se did not escalate the risk of end-stage kidney disease (ESKD) in patients with advanced CKD [12]. These studies were largely cross-sectional and focused on the clinical epidemiology of AKI and CKD amongst COPD patients. Also, outcomes reported in these studies are largely respiratory-related outcomes and early deaths, and the long-term renal impact of AECOPD has not been fully elucidated. Indeed, previous studies suggested that complex pulmonary-renal interactions in chronic lung and kidney conditions [13]. AECOPD can incur excessive airway and systemic inflammatory burden, and hence was postulated to accelerate kidney disease progression [13]. In view of these knowledge gaps, this study was set forth to investigate the impact of AECOPD on long-term renal outcomes and the associated risk factors.

## Methods

We retrospectively reviewed the medical records of all patients who were followed up at the Respiratory Out-patient Clinics in Queen Mary Hospital, Hong Kong in 2015, and identified all COPD patients who had baseline spirometry performed and follow up progress was available in the subsequent five years were included. Patients with co-existing asthma, asthma-COPD overlap, lost to follow-up or those who did not have spirometry or regular renal function assessment were excluded. Clinical and laboratory data were retrieved from the electronic patient records (ePR) of the Hospital Authority. The study was approved by the Institutional Review Board (IRB number: UW 23–356). Patient informed consent was waived as it was a retrospective study without active patient recruitment, and all retrieved clinical data were de-identified. The study was conducted in compliance with the Declaration of Helsinki. Patient data was maintained with confidentiality throughout the study.

AECOPD was defined as an event characterized by dyspnea and/or cough and sputum that worsens over ≤ 14 days [14, 15]. Mild exacerbations were defined in patients treated with short acting bronchodilators (SABD) only. Moderate exacerbations were defined in patients who were treated with SABDs and oral corticosteroids ± antibiotics. Severe exacerbations were defined in patients that required hospitalizations or visit to the emergency room [14]. The clinical/renal outcomes were compared between patients with hospitalized AECOPD in the past one year (1/1/2014 to 31/12/2014) (HAE group) and patients without hospitalized AECOPD in the past one year (1/1/2014 to 31/12/2014) (non-HAE group).

The primary outcome was the development of renal progression or death from all causes upon follow up, starting from 1st January 2015. The secondary outcome was the development of AKI after AECOPD and the time to AKI. Renal progression was defined as decrease in estimated glomerular filtration rate (eGFR) of more than 30 mL/min/1.73 m2 [16]. AKI was defined as increase in serum creatinine (Cr) by ≥ 26.5 µmol/L within 48 h, or increase in serum Cr to ≥ 1.5 times baseline, which is known or presumed to have occurred within the prior seven days [17].

### Statistical analysis

Categorical variables were expressed as frequency and percentage and compared with Chi-square tests or Fisher’s Exact tests. Continuous variables were expressed as mean (± standard deviation [S.D.]) and compared with Student’s t-tests or Mann Whitney U tests. The relationships between hospitalized AECOPD and adverse renal outcomes were first assessed by univariate analysis, followed by multi-variate analysis adjusted for potential confounders including age, gender, baseline forced expiratory volume in one second (FEV_1_), baseline eGFR, baseline Charlson comorbidity index (CCI) and other factors that were significantly different at baseline. Cox regression analysis was used to assess time to AKI. Kaplan–Meier analysis was used to estimate the cumulative progression and death rates and the stratified log-rank statistics to assess the effects of history of hospitalized AECOPD in the past one year with respect to the composite end point. The statistical significance was determined at the level of *p* = 0.05 at two-sided test. All the statistical analyses were done using the 28th version of SPSS statistical package.

## Results

### Patient characteristics

371 patients with COPD were included (Table 1). 309 (83.3%) were males, with a mean age of 80.5 ± 9.0 years. 169 (45.6%) patients had hospitalized AECOPD in the past one year (HAE group) while 202 (54.4%) did not (non-HAE group). The mean baseline eGFR in year 2014, which was checked at clinical stable state in out-patient setting, were 110.4 ± 59.3 mL/min/1.73m^2^ and 96.9 ± 49.4 mL/min/1.73m^2^ in the HAE and non-HAE groups respectively. The baseline CCI were 5.43 ± 1.99 and 5.07 ± 2.15 in the HAE and non-HAE groups respectively. The mean durations of follow-up were 40.1 ± 17.2 months, 37.1 ± 17.4 months and 38.7 ± 17.3 months for HAE group, non-HAE group and the whole cohort respectively.

**Table 1:** Baseline clinical characteristics of patients with chronic obstructive pulmonary disease with or without hospitalized exacerbation in the past 1 year

	Non-HAE group (n = 202)	HAE group (n = 169)	Whole cohort (n = 371)	*p*-values^
Age (years)	81.4 ± 8.9	79.5 ± 9.1	80.5 ± 9.0	0.049*
Gender				0.728
Male	167 (82.7%)	142 (84.0%)	309 (83.3%)	
Female	35 (17.3%)	27 (16.0%)	62 (16.7%)	
Smoking status				0.186
Current smoker	69 (34.2%)	69 (10.8%)	138 (37.2%)	
Former smoker	133 (65.8%)	100 (59.2%)	233 (62.8%)	
Co-morbidities				
Hypertension	194 (96.0%)	159 (94.1%)	353 (95.1%)	0.382
Diabetes mellitus	49 (24.3%)	38 (22.5%)	38 (22.5%)	0.688
Gout	52 (25.7%)	49 (29.0%)	101 (27.2%)	0.483
Stroke/TIA	35 (17.3%)	19 (11.2%)	54 (14.6%)	0.098
Ischemic heart disease	48 (23.8%)	29 (17.2%)	77 (20.8%)	0.118
Medication	24 (21.6%)	18 (16.4%)	42 (19.0%)	
ACEI/ARB	108 (53.5%)	76 (45.0%)	184 (49.6%)	0.103
ICS	190 (94.1%)	168 (99.4%)	358 (96.5%)	0.005*
Baseline lung function parameters at recruitment				
FEV_1_ (L)	1.06 ± 0.42	0.95 ± 0.44	1.01 ± 0.43	0.067
Baseline FEV_1_ (% predicted)	52.0 ± 20.0	43.1 ± 17.9	47.8 ± 19.5	< 0.001*
Baseline FVC (L)	2.20 ± 0.71	2.08 ± 0.74	2.15 ± 0.73	0.247
Baseline FVC (% predicted)	78.8 ± 24.5	71.3 ± 22.5	75.4 ± 23.8	0.082
Baseline FEV_1_/FVC ratio	49.9 ± 14.3	49.8 ± 16.2	48.4 ± 15.3	0.144
Bronchodilator reversibility (mL)	112 ± 108	94 ± 100	104 ± 105	0.282
Bronchodilator reversibility (%)	13.8 ± 14.7	11.3 ± 12.8	12.6 ± 13.9	0.289
Baseline laboratory parameters				
Eosinophil count (x cells/µL)	237 ± 232	248 ± 239	241 ± 235	0.679
Eosinophil %	3.36 ± 3.27	3.40 ± 3.14	3.38 ± 3.21	0.904
Neutrophil count(x10^9^/L)	4.31 ± 1.64	4.70 ± 1.89	4.48 ± 1.75	0.079
Lymphocyte count (x10^9^/L)	1.45 ± 0.73	1.47 ± 0.61	1.46 ± 0.68	0.849
Serum Cr (µmol/L)	83.8 ± 49.0	71.6 ± 27.2	78.2 ± 40.9	0.004*
eGFR (mL/min/1.73m^2^)	96.9 ± 49.4	110.4 ± 59.3	103.0 ± 54.5	0.019*
Charlson co-morbidity index	5.43 ± 1.99	5.07 ± 2.15	5.12 ± 2.04	0.197
Serum albumin level (g/L)	27.0 ± 5.7	26.5 ± 4.5	26.8 ± 5.2	0.386
Serum LDL level (mmol/L)	1.99 ± 0.73	2.05 ± 0.75	2.02 ± 0.73	0.483
Serum urate level (µmol/L)	362 ± 120	330 ± 115	345 ± 118	0.095
HbA1C (%)	5.5 ± 1.9	5.2 ± 2.1	5.36 ± 1.96	0.091
Influenza vaccination	105 (52.0%)	104 (61.5%)	209 (56.3%)	0.081
Pneumococcal conjugate vaccine	15 (7.4%)	14 (8.3%)	29 (7.8%)	0.759
Pneumococcal polysaccharide vaccine	25 (12.4%)	22 (13.0%)	47 (12.7%)	0.853

ACEI, angiotensin converting enzyme inhibitor; ARB, angiotensin receptor blockers; FEV1, forced expiratory volume in 1 s; FVC, forced vital capacity; eGFR, estimated glomerular filtration rates; HAE, hospitalized acute exacerbation in the past 1 year; ICS, inhaled corticosteroids; TIA, transient ischaemic attack

Data expressed as mean ± S.D.

### AECOPD and long-term renal outcomes

285 patients (76.8%) had renal progression/death, which occurred at 37.0 ± 1.5 months after AECOPD. Patients who had developed renal progression/death had longer duration of COPD (7.05 years vs. 4.79 years, *p* = 0.029), higher rates of requiring long-term oxygen therapy (26.2% vs. 7.1%, *p* = 0.024), lower baseline blood eosinophil count and percentage (233 cells/µL vs. 343 cells/µL and 3.30% vs. 4.43%; *p* = 0.033 and 0.039 respectively), and lower serum albumin level (26.6 g/L vs. 29.4 g/L, *p* < 0.001). There was trend to suggest patients with renal progression/death had lower FEV_1_ (% predicted) (47.1% vs. 55.8%, *p* = 0.060) and lower baseline blood lymphocyte count (1.43 × 10^9^/L vs. 1.79 × 10^9^/L, *p* = 0.111), though did not reach statistical significance.

Patients who developed renal progression/death also showed significantly higher annual frequency of AECOPD and hospitalized AECOPD in the past 3 years (2012–2014) [0.97 episodes/year vs. 0.42 episodes/year and 0.89 episodes/year vs. 0.36 episodes/year respectively, *p* = 0.029 and 0.022].

The eGFR and serum Cr level at baseline and their longitudinal changes were summarized in Supplementary Table 1. The HAE group showed a significantly more rapid decline in eGFR than the non-HAE group (-4.64 mL/min/1.73m^2^/year vs. -2.40 mL/min/1.73m^2^/year, *p* = 0.025). Univariate logistic regression revealed that the HAE group had significantly higher chance to develop renal progression or death at 5 years (OR of 2.028 95% CI 1.221–3.369, *p* = 0.006. Hospitalized AECOPD frequency in the past 3 years (2012–2014), any AECOPD in past 3 years and any hospitalized AECOPD in past 3 years (2012–2014) also showed positive correlations with increased risks of renal progression or death in 5 years with [OR 1.367 (95% CI = 1.047–1.785), 2.035 (95% CI = 1.246–3.324) and 2.195 (95% CI = 1.345–3.582) respectively; *p* = 0.022, 0.005 and 0.002 respectively].

Multi-variate analysis showed that patients in the HAE group had significantly higher risk for renal progression/death at 5 years [adjusted OR (aOR) in the HAE group was 2.380 (95% CI = 1.144–4.954), *p* = 0.020] after adjustment for age, gender, baseline FEV_1_, baseline eGFR, baseline CCI, inhaled corticosteroid (ICS) use at baseline, prior exposure of nephrotoxic antibiotics in past 3 years and AECOPD frequency in the follow up period. A possible dose-response relationship was also noted as the frequency of hospitalized HAE in the past 1 year was found to be associated with increased risks of renal progression/death at 5 years with OR of 1.250 (95% CI = 1.025–1.524, *p* = 0.027) and aOR of 1.456 (95% CI = 1.107–1.916, *p* = 0.007) at multivariate analysis.

The frequency of hospitalized AECOPD in the past 3 years, any AECOPD in the past 3 years (2012–2014), hospitalized AECOPD in the past 3 years (2012–2014) remained robust predictors for renal progression or death at 5 years after multivariate analysis [aOR were 1.176 (95% CI = 1.038– 1.331), 2.998 (95% CI = 1.438–6.250) and 2.887 (95% CI = 1.409–5.917) respectively; *p* = 0.011, 0.003 and 0.004].

**Fig. 1:**
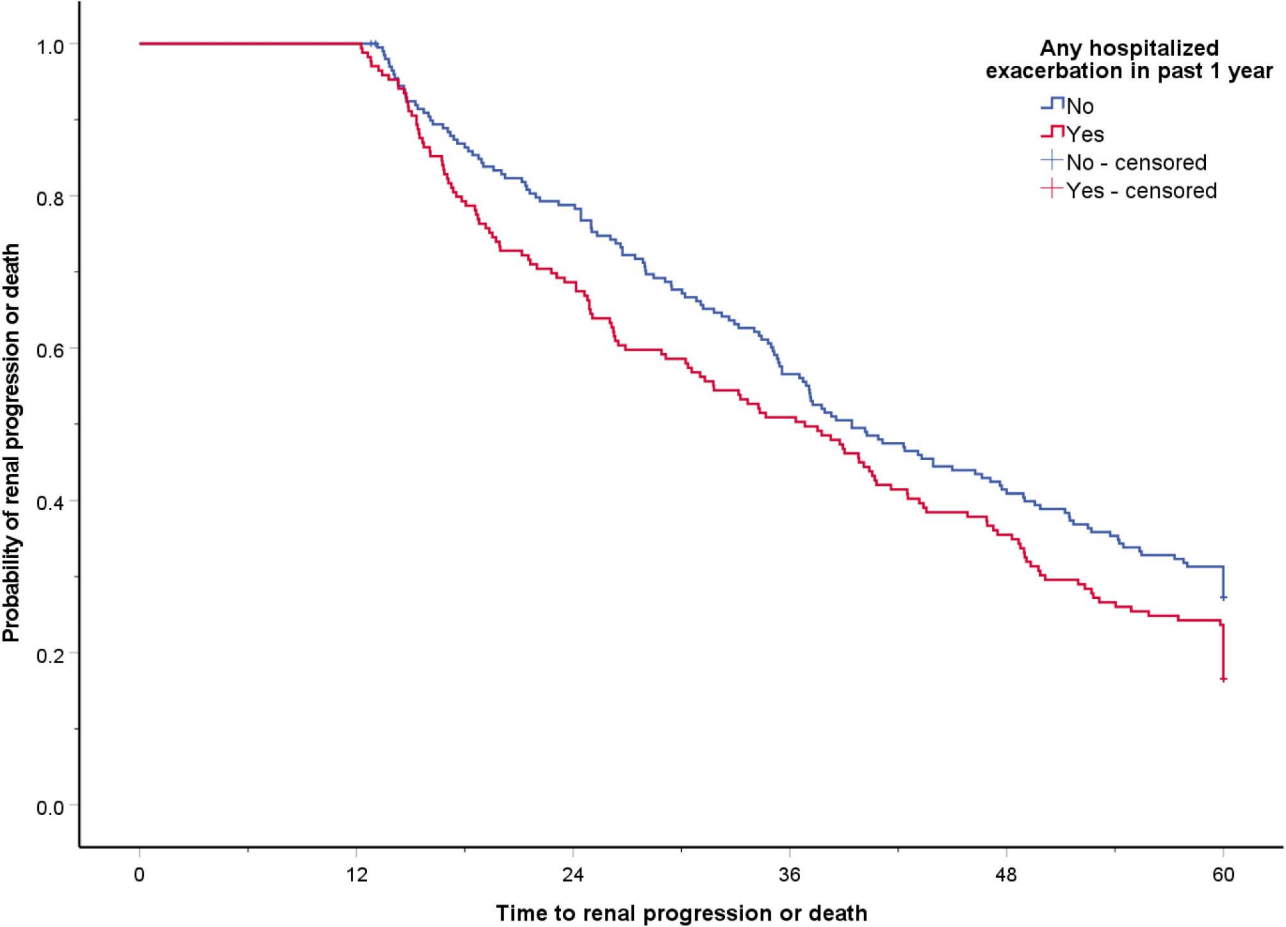
Risk of renal progression or death in chronic obstructive pulmonary disease patients with or without hospitalized acute exacerbation in the past one year

Patients in HAE group also had shorter time to renal progression/death than patients in non-HAE group, with hazard ratio (HR) of 1.317; 95% CI = 1.044–1.661, *p* = 0.020. Multivariate analysis revealed that patients in the HAE group had significantly higher risk of renal progression/death [adjusted HR (aHR) 1.601; 95% CI = 1.141–2.247, *p* = 0.006] after adjusted for age, gender, baseline FEV_1_, baseline eGFR, baseline CCI, ICS use at baseline, prior exposure of nephrotoxic antibiotics in past 3 years and AECOPD frequency in the follow up period. (Fig. 1).

### Hospitalized AECOPD and AKI

102 (27.5%) patients developed AKI in this study. Patients who developed AKI showed numerically lower baseline FEV_1_ (% predicted) [ 51.7 vs. 46.4%, *p* = 0.095], higher annual frequency of AECOPD in past 3 years [1.00 /year vs. 0.89/year, *p* = 0.430], higher annual frequency of hospitalized AECOPD in past 3 years [0.92/year vs. 0.82/year, *p* = 0.436].

**Fig. 2:**
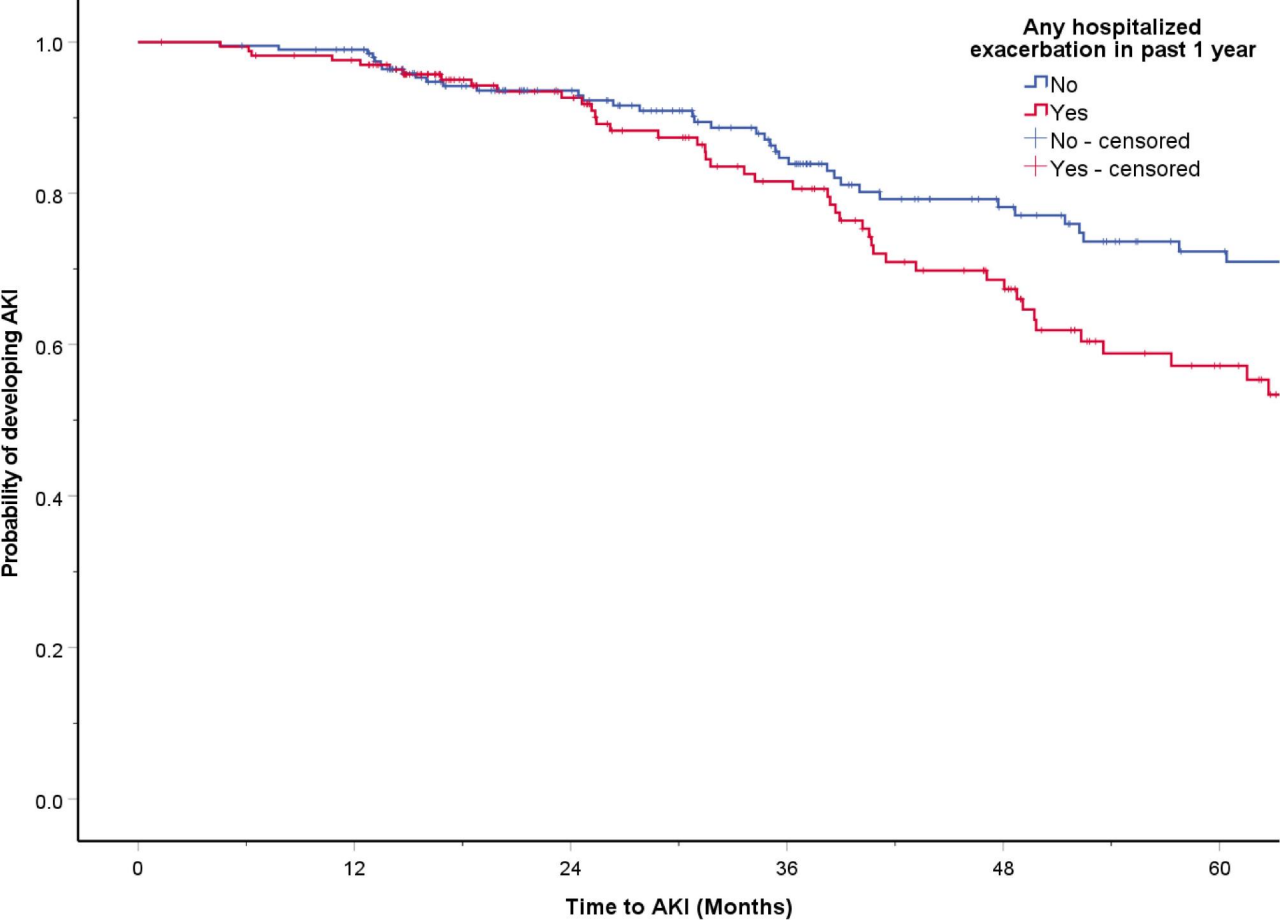
Risk of acute kidney injury in chronic obstructive pulmonary disease patients with or without hospitalized acute exacerbation in the past one year

Patients in HAE group showed an increased risk of AKI compared with patients in the non-HAE group [HR 1.481; 95% CI = 1.002–2.188, *p* = 0.049]. Multivariate analysis further revealed that patients in the HAE group had significantly higher risk of AKI [aHR 2.430; 95% CI = 1.306–4.519, *p* = 0.005] after adjusted for age, gender, baseline FEV_1_, baseline eGFR, baseline CCI, ICS use at baseline and prior exposure of nephrotoxic antibiotics in past 3 years (Fig. 2). Patients who had ≥ 2 moderate exacerbation in past 1 year, those with hospitalized AECOPD in past 3 year, those with ≥ 2 moderate exacerbations /year and those with ≥ 1 hospitalized exacerbation/year in the past 3 years all showed shorter time to AKI (Table 2; Supplementary Figures S1-S4).

**Table 2:** The relationship between acute exacerbations of COPD and development of acute kidney injury

	HR	95% C.I.	*p*-value	aHR^†^	95% C.I.	*p*-value
Any hospitalized exacerbation in past 1 year	1.481	1.002 − 2.188	0.049*	2.430	1.306–4.519	0.005*
2 moderate exacerbations past 1 year	2.108	1.271–3.495	0.004*	2.422	1.128–5.199	0.023*
Any hospitalized exacerbation in past 3 years	1.555	1.030–2.347	0.036*	2.226	1.130–4.384	0.021*
At least 2 moderate exacerbations per year	1.733	1.024–2.931	0.040*	2.040	1.369–3.038	< 0.001*
At least 1 hospitalized exacerbation per year in past 3 years	1.593	1.075–2.362	0.020*	1.769	1.033–3.030	0.038*

*Factors that are statistically significant after adjustment for confounders

^†^Adjustment done for confounders including age, gender, base, baseline forced expiratory volume in 1 s (FEV1), baseline eGFR, baseline CCI, and use of inhaled corticosteroid at baseline

## Discussion

The relationship between COPD and adverse renal outcomes remains elusive. Our study suggested that AKI and progressive CKD were highly prevalent amongst COPD patients, and AECOPD is associated with adverse renal outcomes, both the renal progression and the subsequent development of AKI. Our results highlight the importance of preventing AECOPD, which may translate into improved outcomes in other organ systems including the kidneys. It is crucial to have regular monitoring of kidney function in COPD patients and institute reno-protective treatments if there are early signs of renal deterioration.

In this study, we observed high rates of renal progression/death amongst COPD patients, up to 70%. This is alarming as most patients in this cohort had relatively good renal function at baseline, and renal progression occurred at approximately 3 years from their baseline. Further analysis also identified important patient characteristics which were associated with unfavorable renal outcomes in COPD patients. In this context, patients who showed renal progression had more severe COPD at baseline. Furthermore, COPD patients with non-eosinophilic phenotype appeared to show higher rates of renal progression. Also, patients with poor nutritional status were more likely to develop renal progression/death. More importantly, our results demonstrated that AECOPD and the frequency of exacerbation are robust predictors for renal progression/death. In our study, those patients with history of hospitalized AECOPD in the previous one year, which was an indicator of patients with severe AECOPD [14], were associated with both the development of AKI and renal progression or death from all causes. Indeed, the HAE group in this study showed significantly more rapid decline in renal function compared to the non-HAE group. Patients with a history of hospitalized AECOPD in the previous one year were more likely to have profound respiratory and/or systemic physiological dysfunction and inflammation, often with the needs of treatment with systemic antibiotics and corticosteroids. The adverse long-term renal outcomes in COPD patients who were exacerbation-prone can be explained by the systemic inflammation induced by AECOPD [18]. Our findings call for regular monitoring of kidney function in COPD patients, especially among exacerbators. It is also important to consider reno-protective measures in patients with early evidence of CKD or renal progression.

Apart from long-term renal function decline, our findings also showed that more than one quarter of COPD patients would develop AKI along their disease course. These were in line with previous studies which reported elevated incidence and prevalence of AKI in COPD patients [4, 7, 8, 19]. Unlike progressive CKD, the patient characteristics associated with subsequent AKI are less well defined in this study. Notwithstanding, patients who are exacerbation-prone (i.e., the HAE) group showed a 4-fold increased risk of AKI compared to the non-HAE group. Furthermore, patients with lower FEV_1_ appeared to show higher risk of subsequent AKI and frequency of AECOPD also showed a trend of escalated risk of AKI. Also, the frequency of AECOPD showed a tendency of increased AKI risk. The role of inflammation plays a significant role in the development of AKI [20]. Other possible pathogenic mechanisms of how AECOPD may cause AKI include hypoxemia and hypoperfusion during AECOPD [21, 22], use of nephrotoxic antibiotics for treatment of AECOPD [23], hyperglycemia associated with systemic corticosteroid use [24]. Thus, COPD patients, especially those with frequent AECOPD, are at risk of AKI and it is crucial to avoid hypotension, hypoxaemia and nephrotoxic antibiotics in managing these vulnerable subjects.

In our cohort, more than 90% of the patients were on ICS. There were more patients on ICS in the HAE group. The high percentage of patients treated with ICS in this cohort could be explained by the fact that ICS remained one of the key treatments of choice for COPD back in 2015 [25, 26]. Nonetheless, the higher percentage of patients on ICS in HAE could be related to the fact that these patients were at increased risks of AECOPD, which is consistent with the current recommendation.

The limitations of this study were the single-centre retrospective nature and the relatively small patient number included. The relatively small number of patients in this study was because we only included COPD patients with detailed spirometry results and comprehensive follow-up data (especially renal function assessment), but this have helped ensure data completeness and homogeneity to assess the relationship between COPD severity and renal progression. Being retrospective in nature, the number of renal function tests and the timing of the tests were not unified. Ideally, a protocolized regular monitoring of renal function in a prospective study could avoid this potential selection bias. But as the majority of the patients in the cohort had various co-morbidities, especially hypertension in more than 90% of the patients, they would have at least annual renal function test as part of complication screening and assessment. One should also appreciate that older age and medical co-morbidities may influence the risk of renal progression/death. To address this limitation, we have also included CCI to adjust for the confounding effect of advanced age and medical co-morbidities in our multivariate analysis. Ideally, a separate prospective study on younger COPD patients with longer follow-up interval will better elucidate the relationship between COPD and renal progression. Notwithstanding, due to the healthcare set-up in Hong Kong, most AECOPD patients were admitted to public hospitals and hence our results provided an unbiased real-world data on the short- and long-term renal outcomes in COPD patients (e.g. elderly COPD patients with multiple co-morbidities). Moreover, COPD patients in our catchment area were managed and followed at a designated respiratory clinic, in which the chronic management were largely standardized, and clinical events were clearly documented. Therefore, our data included comprehensive clinical information as well as lung function and laboratory parameters, thereby allowing us to properly evaluate the impact of COPD severity and AECOPD on short- and long-term kidney outcomes.

Taken together, our present findings provide further evidence of the adverse non-respiratory outcomes from AECOPD. AECOPD is not only related to AKI, but is also associated with long-term renal dysfunction, which is irreversible. While the consequences of acute and/or chronic renal insufficiency increase the burden of COPD patients, they also confer negative impacts on various clinical aspects, for example, limiting the options of pharmacotherapy for co-morbidities, aggravating cardiovascular complications, increasing healthcare service utilization, and worsening patient survival. As such, timely initiation of preventive measures after an episode of AECOPD should not be over-emphasized as the benefits may extend beyond optimizing respiratory conditions, but also other systemic morbidity, including renal and cardiovascular outcomes.

## Conclusions

AKI and CKD are highly prevalent in COPD patients, with AECOPD being a robust risk factor for renal progression/death and AKI. Prevention of AECOPD may potentially optimize short- and long-term renal outcomes.

### Electronic supplementary material

Below is the link to the electronic supplementary material.

Supplementary Material 1

## Data Availability

All available data are presented in the manuscript and no additional data will be provided. Data is not available to be shared.

## Declarations

### Clinical trials

This study is not a clinical trial, and no necessary registration details will be provided.

### Notation of prior abstract publication/presentation

Nil.
