# Supplementary material for: Hospitalized acute exacerbation in chronic obstructive pulmonary disease – impact on long-term renal outcomes
Source: Respir Res. 2024 Jan 18;25:36. doi: 10.1186/s12931-023-02635-8 (PMC10797933; doi:10.1186/s12931-023-02635-8)

**Supplementary Table**

**ST1 Serum creatinine and eGFR levels in patients who have or have not developed hospitalized acute exacerbations**

|  | | **Baseline** | | **Two Years** | | | **Three Years** | | | **Five Years** | |
| --- | --- | --- | --- | --- | --- | --- | --- | --- | --- | --- | --- |
|  |  | No HAE | HAE | No HAE | HAE | | No HAE | | HAE | No HAE | HAE |
| **Cr (µmol/L)** | 83.8±49.0 | | 71.6±27.2 | 101.8±75.6 | 90.3±46.2 | 99.9±49.4 | | 92.5±33.0 | | 98.2±44.2 | 104.3±59  .6 |
| **eGFR (mL/min/1.73m^2^)** | 96.9±49.4 | | 110.4±59.3 | 79.2±35.8 | 86.8±41.7 | | 76.6±35.5 | 80.5±38.8 | | 75.7±30.9 | 77.7±35.3 |

Cr = Creatinine, eGFR = Estimated glomerular filtration rate, HAE = Patients had hospitalized AECOPD in past 1 year, No HAE = Patients without hospitalized AECOPD in past 1 year

**Supplementary Figures**

**SF1. Risk of acute kidney injury in chronic obstructive pulmonary disease patients with or without 2 moderate exacerbations in the past 1 year**

**
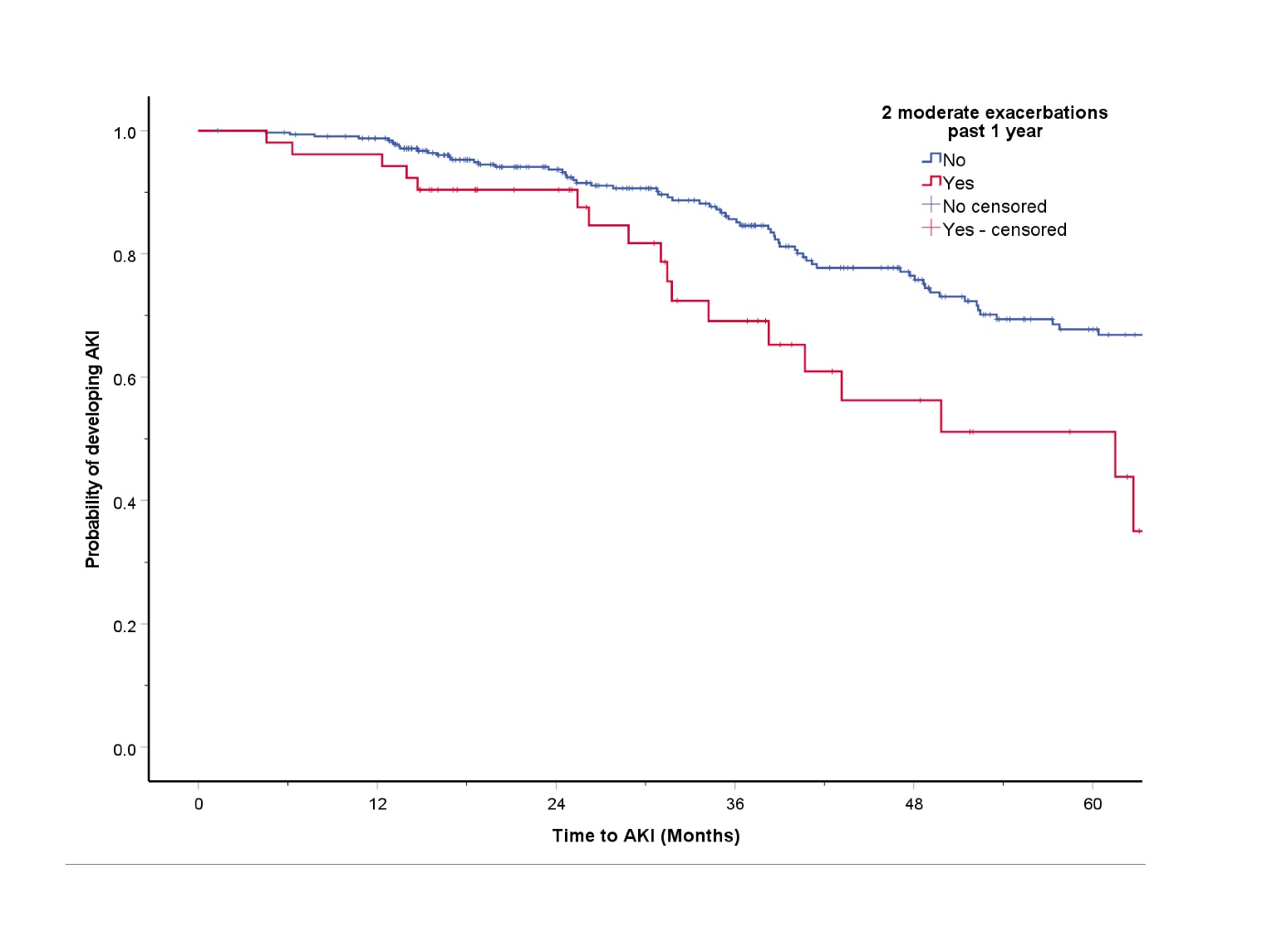
**

**SF2. Risk of acute kidney injury in chronic obstructive pulmonary disease patients with or without hospitalized acute exacerbation in the past three years**

**
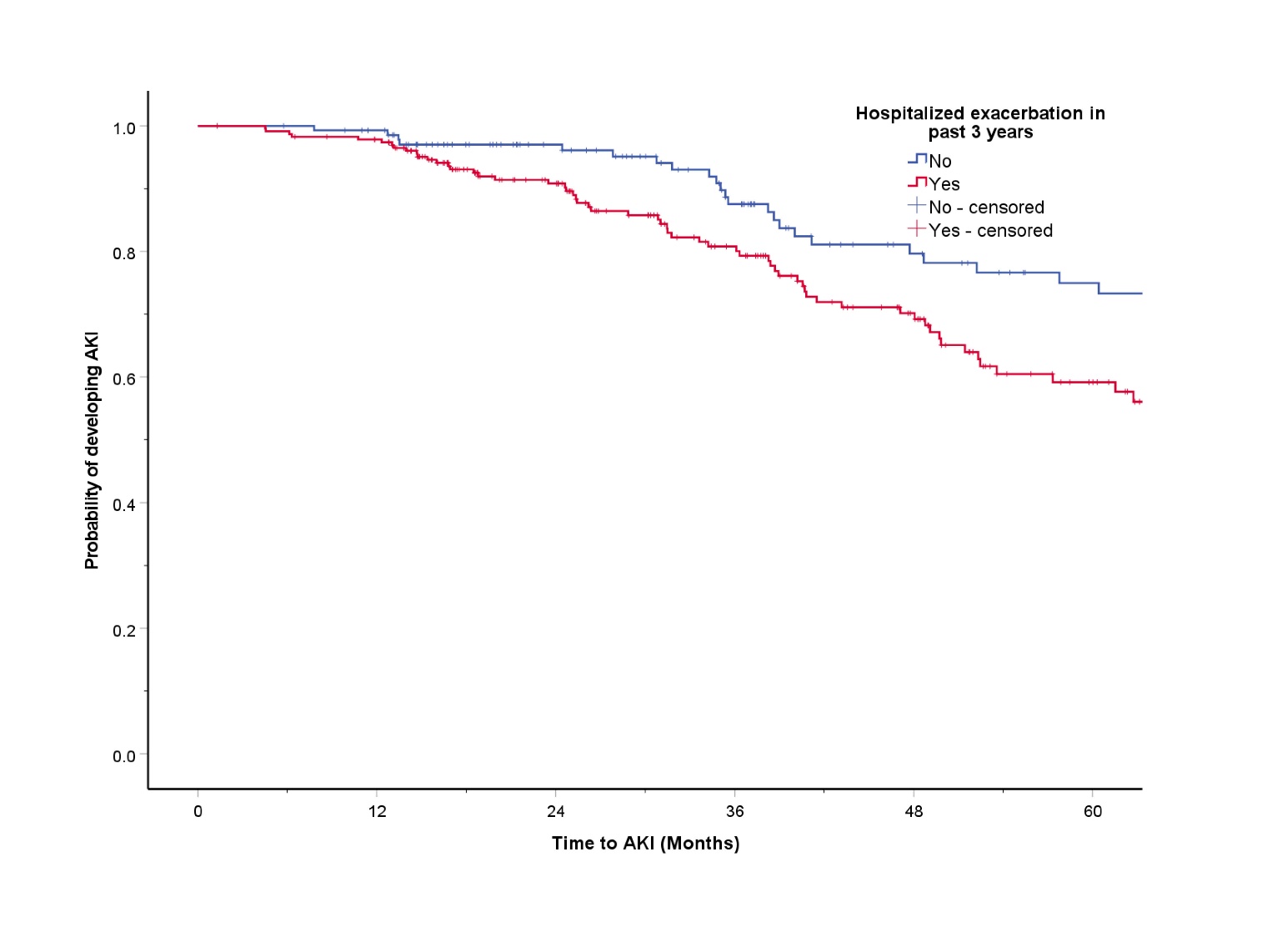
**

**SF3. Risk of acute kidney injury in chronic obstructive pulmonary disease patients with or without at least 2 moderate exacerbations per year**

**
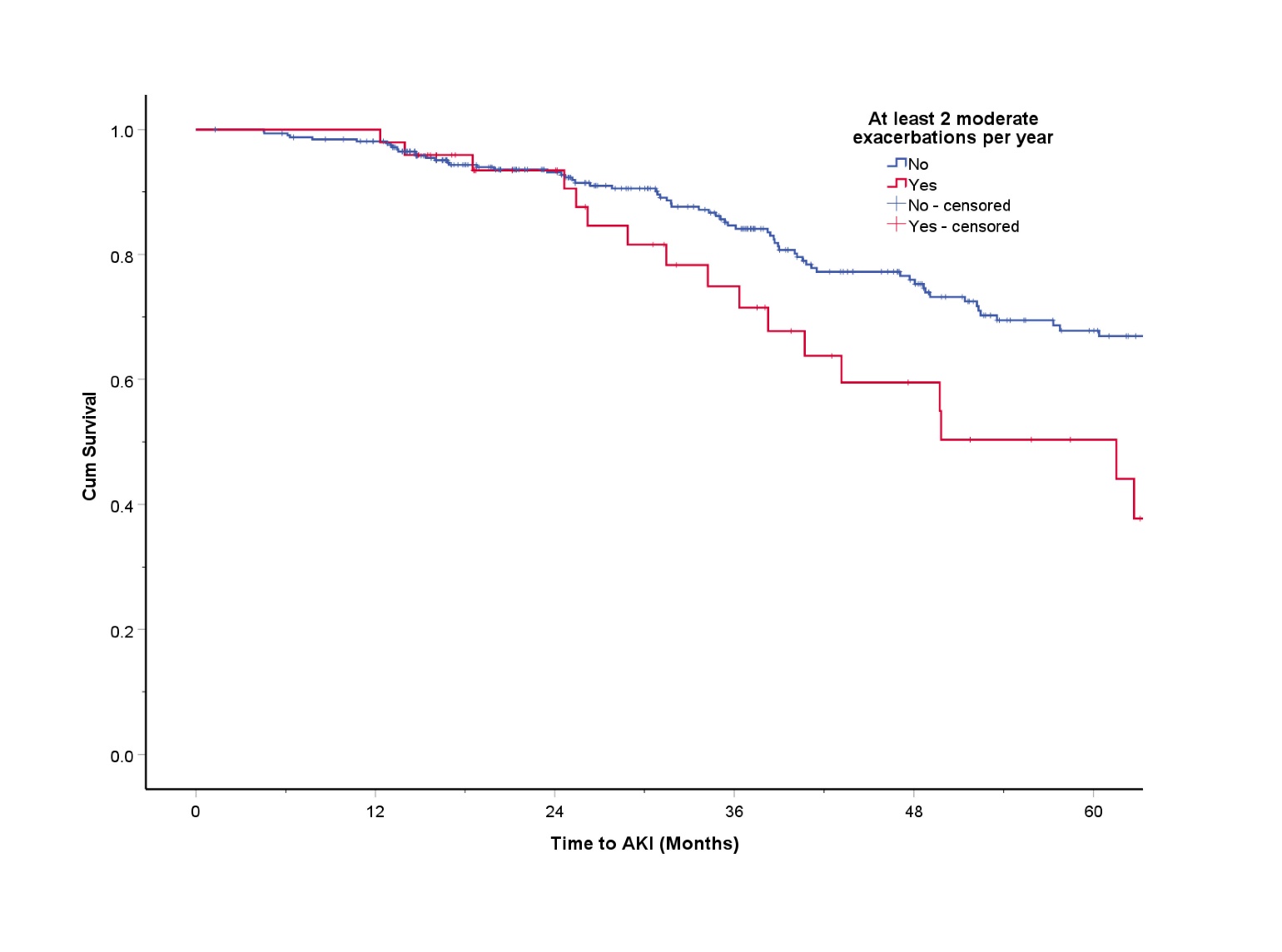
**

**SF4. Risk of acute kidney injury in chronic obstructive pulmonary disease patients with or without at least 1 hospitalized exacerbation per year in past 3 years**


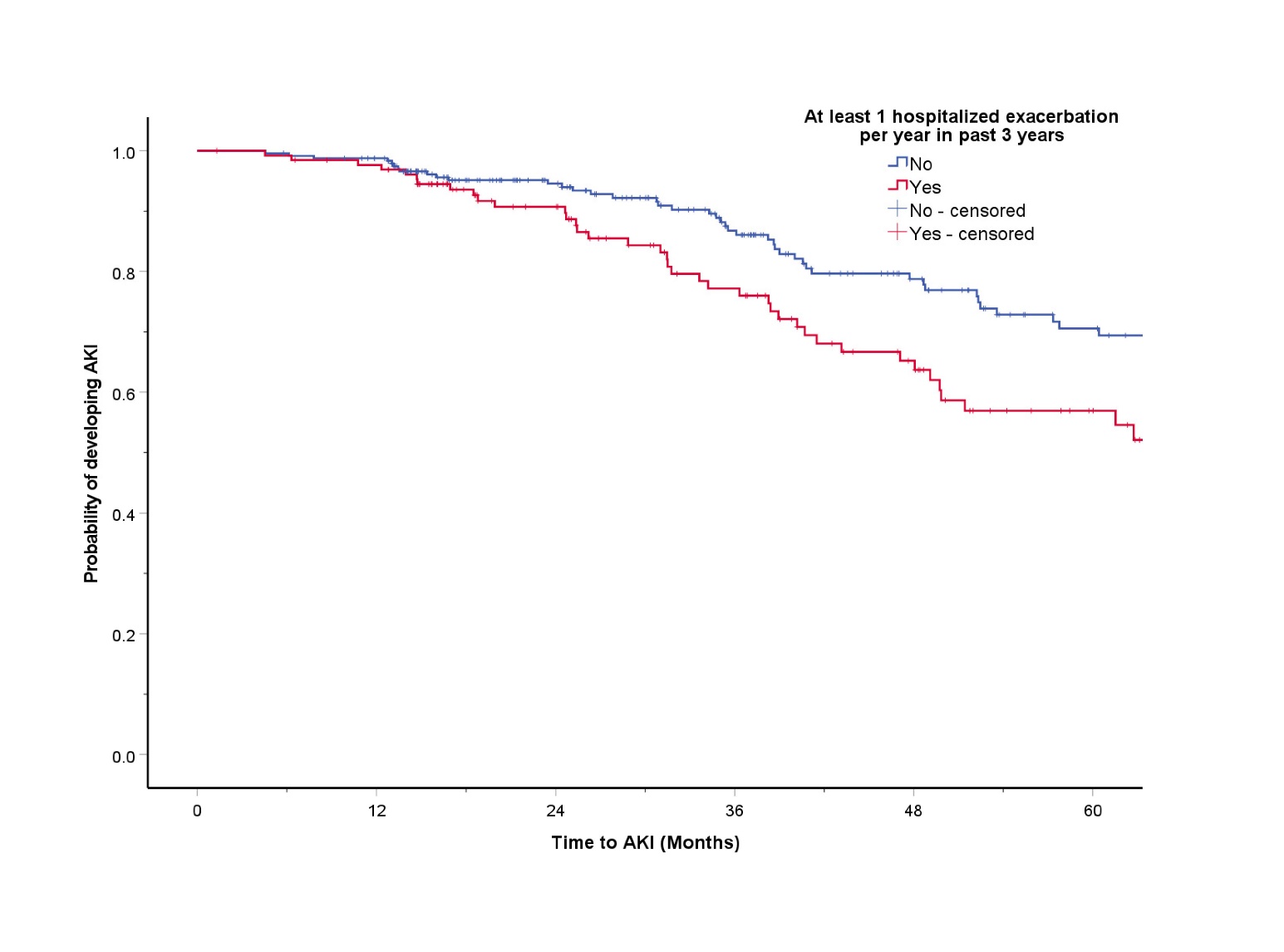

Supplement: Supplementary file 1 — Supplementary Material 1 [file 12931_2023_2635_MOESM1_ESM.docx]
